# Commentary on “Shifting Paradigm: Utilization and Outcomes with Neoadjuvant Chemotherapy for cT4 and cN2 Colon Cancers”

**DOI:** 10.3390/cancers18091487

**Published:** 2026-05-06

**Authors:** Carlo Alberto Schena, Luigi Marano, Sergio Alfieri, Fausto Rosa

**Affiliations:** 1 Department of General Surgery, Fondazione Poliambulanza Istituto Ospedaliero, 25124 Brescia, Italy; 2 Department of Medicine, Academy of Applied Medical and Social Sciences-AMiSNS, Akademia Medycznych i Spolecznych Nauk Stosowanych, 82-300 Elblag, Poland; l.marano@amisns.edu.pl; 3 Department of General Surgery and Surgical Oncology, “Saint Wojciech” Hospital, “Nicolaus Copernicus” Health Center, 80-462 Gdansk, Poland; 4 Department of Translational Medicine and Surgery, Facoltà di Medicina e Chirurgia, Università Cattolica del Sacro Cuore, 00168 Rome, Italy; sergio.alfieri@policlinicogemelli.it; 5 Department of Surgery, Fondazione Policlinico Universitario Gemelli IRCCS, 00168 Rome, Italy

We find the article by Hartley et al. on neoadjuvant chemotherapy (NAC) for cT4 and cN2 mismatch repair-proficient colon cancers very interesting, in which the authors report improved R0 resection rates, a lower lymph node ratio, and better overall survival, particularly in cT3N2 and cT4N2 disease [1]. This large NCDB-based analysis provides valuable real-world data for an evolving field. However, before embracing NAC as a true “shifting paradigm” for high-risk colon cancer, we believe three aspects merit further clarification and development.

First, the authors adopt a per-protocol approach by excluding patients who did not proceed to surgery after NAC, acknowledging that this may bias survival in favor of the NAC cohort. Yet, neither the proportion nor the clinical profile of these non-operated patients is reported, and their outcomes remain unknown. We recognize that the NCDB, as a retrospective administrative database, may not capture granular clinical data such as documented chemotherapy toxicity, radiological evidence of disease progression, or formal performance status assessments. Nevertheless, at minimum, we would argue that reporting the proportion of NAC patients who never reached resection, alongside available demographic proxies such as age, Charlson comorbidity index, and facility type, all fields accessible within the NCDB, would provide the missing denominators that are most clinically relevant. In a population that is often elderly and comorbid, the risk that frail patients may experience toxicity, progression, or functional decline during NAC is clinically meaningful [2,3]. From a decision-making standpoint, quantifying the number of patients who never reach resection and characterizing the age, comorbidities, and performance status in this subgroup is crucial for balancing the survival gains observed among “treatment completers” against the potential harm in vulnerable individuals.

Second, Hartley et al. document an impressive concordance between clinical and pathological staging, with over 90% agreement for T4 and N2 status and less than 2% of cN2 cases eventually found to be pN0. These data support using clinical T4/N2 as a trigger for NAC in the NCDB setting [4]. However, the generalizability of this high staging accuracy outside accredited NCDB centers remains uncertain. Published data from the international FOXTROT trial setting have documented substantial variability in CT staging performance when moving from highly controlled, protocol-driven environments to broader, real-world conditions [5,6]. In routine practice, differences in CT acquisition protocols, reporting standards, and radiologic expertise may lead to both overstaging and understaging, particularly for nodal disease [5,6]. Beyond highlighting this limitation, we suggest that structured multidisciplinary teams, integrating radiologists with dedicated gastrointestinal imaging expertise, medical oncologists, and surgeons, represent a potentially actionable mechanism to reduce inter-institutional variability in clinical staging, even in lower-volume or non-NCDB settings [7]. Future research could specifically examine how NAC performs in lower-volume or non-NCDB institutions, where clinical staging may be less accurate and the risk of treating overstaged disease with NAC is not negligible.

Third, and beyond the methodological remarks above, we wish to highlight what we consider an important translational opportunity that the neoadjuvant setting uniquely offers, one which is not a limitation of the original study, whose retrospective NCDB design appropriately precluded such analyses, but rather a perspective for how future prospective trials might capitalize on this therapeutic window. The study by Hartley et al. alludes briefly to ctDNA in the context of early metastatic dissemination; however, cT4/cN2 colon cancer represents an ideal translational platform for response-adapted perioperative strategies. Serial ctDNA measurements before, during, and after NAC could help distinguish patients in whom systemic therapy converts aggressive biology into durable control from those with persistent molecular disease who may require escalation, novel agents, or alternative treatment paths [8,9,10]. Similarly, integrating tissue-based molecular profiling and immune signatures into ongoing and future randomized trials could enable dynamic tailoring of NAC duration, postoperative adjuvant intensity, and surveillance, moving beyond a “one-size-fits-all” sequence.

In summary, Hartley et al. provide robust real-world evidence supporting NAC as a promising strategy for selected cT4 and cN2 colon cancers. To justify a genuine paradigm shift, we believe future studies should quantify and characterize non-operated NAC patients, test the reproducibility of high clinical–pathological concordance in less controlled environments, potentially leveraging MDT-based staging protocols as a bridge toward standardization, and fully exploit the neoadjuvant setting as a biomarker-driven, ctDNA-informed platform for precision perioperative care.
